# Bilateral Simultaneous Optic Neuritis Following Envenomations by Indian Cobra and Common Krait

**DOI:** 10.3390/toxins14110805

**Published:** 2022-11-19

**Authors:** Subramanian Senthilkumaran, Stephen W. Miller, Harry F. Williams, Ponniah Thirumalaikolundusubramanian, Ketan Patel, Sakthivel Vaiyapuri

**Affiliations:** 1 Manian Medical Centre, Erode 638001, Tamil Nadu, India; 2 The Poison Control Center, Children’s Hospital of Philadelphia, Philadelphia, PA 19104, USA; 3 Toxiven Biotech Private Limited, Coimbatore 641042, Tamil Nadu, India; 4 Department of General Medicine, The Tamil Nadu Dr M.G.R Medical University, Chennai 600032, Tamil Nadu, India; 5 School of Biological Sciences, University of Reading, Reading RG6 6UB, UK; 6 School of Pharmacy, University of Reading, Reading RG6 6UB, UK

**Keywords:** snakebite envenomation, Indian cobra, common krait, optic neuritis, corticosteroids

## Abstract

In India, most snakebite envenomation (SBE) incidents are caused by the “Big Four” snakes which include Russell’s viper, common krait, Indian cobra, and saw-scaled viper. Their common envenomation effects include neurotoxicity, myotoxicity, and coagulopathy. However, they also induce rare complications such as priapism, pseudoaneurysm, and sialolithiasis. Ocular manifestations such as optic neuritis develop rarely following envenomations by non-spitting snakes and they may cause temporary vision changes and blindness if untreated. While optic neuritis following Indian cobra envenomation has been reported previously, this was not encountered in victims of common kraits. Hence, for the first time, we report optic neuritis developed in a victim following envenomation by a common krait and compare its clinical features and diagnostic and therapeutic methods used with another case of optic neuritis in a victim of an Indian cobra bite. Both patients received antivenom treatment and made an initial recovery; however, optic neuritis developed several days later. The condition was diagnosed using ophthalmic examination together with computed tomography and/or magnetic resonance imaging methods. Due to very similar clinical features, both patients received intravenous corticosteroids which restored their vision and successfully treated optic neuritis. This case report suggests that the optic neuritis developed in a common krait envenomation is comparable to the one developed following a cobra bite, and therefore, the same diagnostic and therapeutic approaches can be used. This study also raises awareness of this rare complication and provides guidance for the diagnosis and treatment of SBE-induced optic neuritis.

## 1. Introduction

Venomous snakebites are common in South and Southeast Asia, Africa, as well as in Central and South America [1]. The significant morbidity and mortality resulting from these injuries are mostly in rural areas that are poorly equipped to handle such medical emergencies [1,2,3,4]. Snakebite envenomation (SBE) causes around 150,000 deaths and 500,000 disabilities worldwide every year [1]. In India alone, around 58,000 deaths occur annually and most of these are attributed to the “Big Four” snake species which include Russell’s viper (*Daboia russelii*), Indian cobra (*Naja naja*), saw-scaled viper (*Echis carinatus*), and common krait (*Bungarus caeruleus*) [5]. SBE victims are often agricultural workers who accidentally encounter snakes while performing their regular activities. The victims mostly lack basic protection such as suitable footwear and gloves which might prevent bites and envenomation [3,6]. The envenomation effects depend on several factors but the species responsible and the amount of venom injected are the key determinants. The most common envenomation effects are neurotoxic, myotoxic, cytotoxic, nephrotoxic, and coagulopathic complications [7]. However, SBE may cause several rare complications such as priapism [8], spleen rupture [9], Wunderlich syndrome [10], pseudoaneurysm [11], and sialolithiasis [12].

Optic neuritis is a frequently observed clinical condition that occurs due to the inflammation of optic nerves [13]. This condition is mostly associated with multiple sclerosis; however, there are several factors such as autoimmune disorders and infections which can also induce optic neuritis [13]. The delay in diagnosing and treating this condition may result in permanent loss of vision. SBE-induced ocular complications are frequently reported, although their clinical pathology, underlying molecular mechanisms, appropriate diagnosis, and treatment methods are not well understood [14]. While most of the reported ocular complications are caused by venoms sprayed directly into the eyes from spitting cobras, the non-spitting snakes (including vipers) are known to induce ocular complications following injection of venom in other body parts such as lower and upper limbs [14]. For example, central retinal artery occlusion (CRAO), acute angle-closure glaucoma (AACG), cataract, corneal oedema, macular infarction, and subconjunctival haemorrhage have been rarely reported in victims following envenomation by vipers or other non-spitting snake species [14,15,16,17]. To the best of our knowledge, common krait-induced optic neuritis has not been reported previously. Hence, we report the clinical presentation, diagnosis, and treatment of optic neuritis that developed in a patient following envenomation by a common krait and compare their features with the one developed in a victim following a cobra bite. Despite antivenom treatment, the ocular manifestations developed in both patients several days after the envenomation. This article provides awareness and approaches for the diagnosis and treatment of optic neuritis induced by common krait envenomation (similar to cobra bites) which will be beneficial for SBE management, especially in rural areas.

## 2. Case Report 1: Indian Cobra-Induced Optic Neuritis 

**Figure 1:**
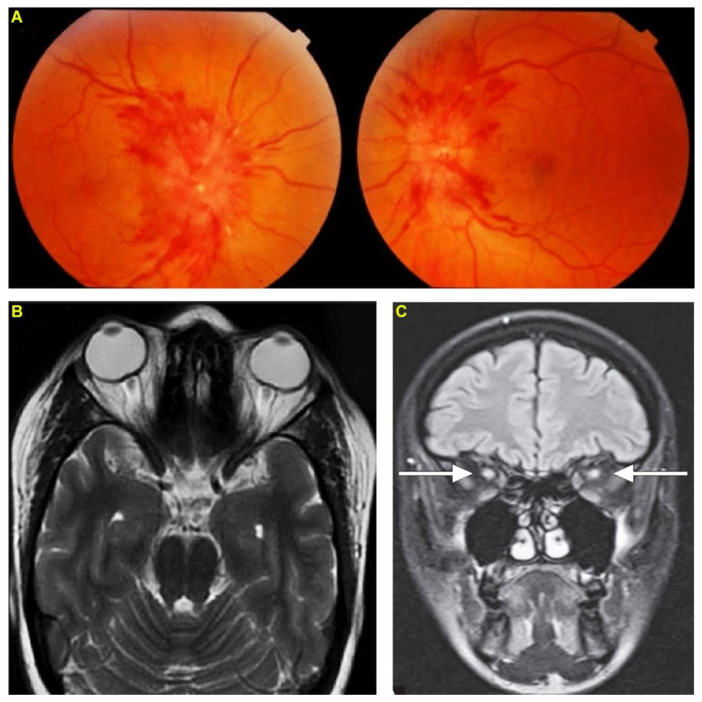
Envenomation by an Indian cobra induces optic neuritis. Patient 1 following a bite from an Indian cobra has developed bilateral papilledema (**A**). (**B**) magnetic resonance imaging orbit shows effacement of the perioptic-optic cerebrospinal fluid space and diffuse increase in T2 signal involving the optic nerve. (**C**) coronal fat-suppressed T2-weighted magnetic resonance imaging shows hypertense thickening of perioptic nerve sheath surrounded by cerebrospinal fluid providing a ‘polo-mint’ appearance (indicated by arrows).

Patient 1, a healthy 32-year-old female homemaker, was bitten by an Indian cobra on her right foot. She developed bilateral ptosis and respiratory depression within 30 min following the bite. After evaluation in a local hospital, she was treated with an intravenous infusion of 10 vials (100 mL) of polyvalent antivenom raised against the Indian ‘Big Four’ snakes as well as neostigmine and atropine. Although she improved, on the fifth day after the bite, she complained of decreased vision in both eyes and therefore was referred to the emergency department of our hospital. The patient arrived at the emergency department without any neurological deficits. However, an ophthalmic examination of both eyes revealed sluggish pupillary reactions to light, papilledema (Figure 1A), and the best-corrected vision was finger counting close to the face in both eyes. Anterior slit lamp examination, intraocular pressure and extraocular motility were all normal. Based on these findings, benign intracranial hypertension was suspected, and therefore, brain computed tomography (CT) imaging with contrast was performed but it showed normal results. Lumbar puncture showed normal opening pressure of cerebrospinal fluid (CSF). Visually evoked responses showed increased latency and decreased amplitude indicative of bilateral optic neuritis. Finally, an MRI of the brain revealed effacement of the perioptic-optic CSF space and a diffuse increase in T2 signal involving both optic nerves on T2-weighted images (Figure 1B). MRI also revealed a hyperintense thickening of the perioptic nerve sheath surrounded by CSF giving a ‘polo mint’ appearance on fat-suppressed T2-weighted images (Figure 1C) confirming the diagnosis of optic neuritis. Based on the clinical features, the patient received three doses of intravenous methylprednisolone (1 g of methylprednisolone over 40 min for each dose) over three days to treat optic neuritis. Following this treatment, the pain subsided rapidly, and the patient was able to see shapes on the following day and her symptoms gradually returned to the baseline vision at 45 days. The patient was monitored regularly for up to six months and no other residual effects were observed in their vision or eyes.

## 3. Case Report 2: Common Krait-Induced Optic Neuritis 

**Figure 2:**
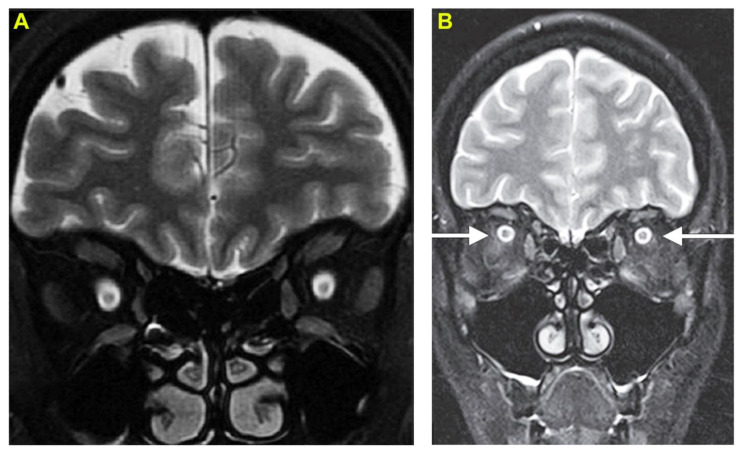
Optic neuritis following envenomation by a common krait. (**A**) coronal T2-weighted FLAIR magnetic resonance imaging shows high signal changes in both the optic nerves. (**B**) coronal T2-weighted magnetic resonance imaging with fat suppression indicates enlarged optic nerves surrounded by cerebrospinal fluid providing a ‘polo-mint’ appearance (indicated by arrows).

Patient 2, a 30-year-old male farmer, was bitten by a common krait while working on his farmland. He was transported to the emergency department within one hour following the bite. He was unconscious and in respiratory distress upon admission. He was immediately intubated and started on mechanical ventilation. He was intravenously administered 20 vials (200 mL) of polyvalent antivenom (Bharat Serum and Vaccines Limited, Mumbai, India) raised against the Indian ‘Big Four’ snakes. His sensorium, muscle strength and oxygen saturation improved and were extubated two days after admission/bite. On the fourth day after the bite, he complained of blurred vision and diminished visual acuity similar to patient 1. So, he underwent a thorough neurological examination. His motor and sensory system examinations were normal without any signs of cerebellar dysfunction or meningeal irritation. His ophthalmic examination was notable for a visual acuity that was limited to the perception of light and round pupils but sluggishly reactive to light. His intraocular pressure was within normal limits and ocular motility and anterior segments were unremarkable. Fundoscopic examination revealed bilateral papilledema. The visual evoked potentials were indicative of maculopathy and bilateral axonopathic optic neuropathy consistent with optic neuritis. MRI of the brain revealed high-intensity changes in both optic nerves on T2-weighted images (Figure 2A) and enlarged optic nerves surrounded by CSF giving a ‘polo mint’ appearance on fat-suppressed T2-weighted images (Figure 2B) supporting the diagnosis of optic neuritis. Like patient 1, this patient also received three doses of intravenous methylprednisolone over three days. Following this treatment, the pain reduced rapidly, and the patient was able to perceive hand movements from the second day of treatment. His vision gradually improved to bilateral visual acuity of 6/60 on the sixth day and 6/6 in both eyes within 30 days. Regular monitoring for up to six months did not reveal any further residual effects in their vision or eyes.

## 4. Discussion

Anatomically, greater than one million axons of each optic nerve connect the rods and cones of each eye to the optic chiasm in the brain [18]. The optic disc serves to connect the axons of the retinal ganglion cells with the optic nerve. The optic nerves are considered an extension of the central nervous system and are divided into four segments which include the intraocular, intraorbital, intracanalicular, and intracranial [18]. The retina and choroid are rich in blood vessels, but the cornea and lens lack vasculature. Optic neuritis commonly refers to a demyelinating condition and is often described in patients with multiple sclerosis [19]. However, this term is also used more broadly to describe other optic neuropathic complications which result from inflammation of the optic nerves [13]. Autoimmune disorders such as systemic lupus erythematosus and Sjogren’s disease, microbial infections, and other local or systemic inflammatory processes are known to induce optic neuritis. The early symptoms of optic neuritis include blurred or diminished vision, but the delay in seeking appropriate treatment may result in permanent loss of vision. 

Although SBE has been known to induce a plethora of local and systemic complications, ocular manifestations are rarely encountered following envenomations, specifically by non-spitting snake species. SBE-induced ocular complications include subconjunctival haemorrhage, AACG, CRAO, acute anterior uveitis, external ophthalmoplegia, exudative retinal detachment, photophobia, macular infarction, and optic neuritis [14]. Several species of spitting cobras including *Naja atra*, *Naja nigricollis*, *Naja pallida*, and *Hemachatus haemachatus* from various geographical locations are known to induce ocular complications including optic neuritis. In many cases, optic neuritis developed a few or several days after envenomation. In some patients, simple water irrigation of the eyes was used to manage ocular damage, while in others, a range of treatment methods was used [14]. While spitting cobras directly spray their venom into the eyes and cause ocular complications, the non-spitting snake species induce complications through the injection of venom in other body parts that may induce direct or indirect effects on the eyes based on their pharmacokinetic behaviour [20]. The impacts of various toxins present in elapid and viper venoms on the ocular system vary based on their molecular mechanisms. Therefore, it is important to thoroughly investigate the ocular complications induced by SBE specifically from non-spitting species to underpin their clinical pathology and develop appropriate diagnostic and therapeutic strategies.

In this study, we report a unique case of optic neuritis that developed in a patient following envenomation from a common krait and compare their clinical features with optic neuritis developed in another patient who was bitten by a cobra, both non-spitting elapid species. In both cases, optic neuritis developed a few days after the bite despite antivenom treatment. The subjective complaints of blurred vision in both scenarios prompted thorough neurological and ophthalmological examinations followed by CT and MRI scans which revealed the development of optic neuritis. Despite envenomations by two different snakes in these patients, the clinical features developed in both were very similar. Moreover, the use of MRI was beneficial in diagnosing optic neuritis and identifying the unique ‘polo-mint’ appearance of enlarged optic nerves in both patients. The envenomations from non-spitting cobras, including the Indian cobra, were previously reported to induce optic neuritis in a few cases. For example, an Indian cobra bite in a 50-year-old male caused diminished visual acuity on the sixth day after SBE and improved over the next few days [21]. This patient displayed sluggish pupils and diminished visually evoked responses although their fundus was normal. The patient was treated with an intravenous infusion of dexamethasone for three days and a tapered oral prednisolone regimen over three weeks with gradual improvement in his vision. Similarly, another case of Indian cobra bite developed optic neuritis six days after the bite despite receiving the antivenom treatment [22]. This patient was treated with intravenous methylprednisolone for seven days followed by a prednisolone taper regimen, which improved their condition. However, a case of optic neuritis developed in a 7-year-old child following suspected envenomation by a Cape cobra resulting in a permanent disability [23]. While there are limited reports available for optic neuritis following non-spitting cobra bites, we were unable to find any reports of optic neuritis following krait envenomation, although they predominately induce neuromuscular effects [24]. Hence, this study reports the first case of optic neuritis developed following krait envenomation, and its clinical features are comparable to cobra-induced optic neuritis. 

In addition to elapid snake species, some vipers such as *Crotalus atrox* (which is known to spit venom in some cases) [25,26], *Daboia russelii* [27], and *Macrovipera lebetinus* [28] as well as specific colubrid snakes were reported to induce optic neuritis [14]. In a Russell’s viper bite victim, bilateral blindness developed within 30 min following the bite and improved upon treatment with methylprednisolone [27]. In contrast, *Macrovipera lebetinus* induced blurred vision and optic neuritis in a patient six days after the bite [28]. In two cases of viper bites, the venom injection occurred directly in the eyes of the patients resulting in severe necrosis and subconjunctival haemorrhage [29,30]. While in some cases, ophthalmological examinations revealed optic neuritis, in others, CT and MRI scans were required to ascertain the development of this condition. As used in this study, we strongly recommend the use of these sophisticated methods to ascertain the precise nature of damage induced by optic neuritis. Moreover, most of the reported optic neuritis cases were treated with intravenous and/or oral administration of corticosteroid drugs. In patients with mild ocular effects, water irrigation, topical prednisolone, and/or specific antibiotics were used to successfully manage this condition. 

There are several potential causes reported for the development of optic neuritis following SBE [14]. For example, haemorrhage (including those distant from the ocular structures), allergic reactions to antivenoms, and capillary or other vascular injuries are likely to induce inflammation and subsequent ocular damage. Snake venom toxins are well known to induce systemic inflammatory responses with leucocytosis and release various inflammatory mediators such as IL-4, IL-6, IL-8, and TNFα [31]. Therefore, this may play a critical role in the development of optic neuritis along with vascular damage and haemorrhage. Venom metalloproteases induce the degradation of extracellular matrix and basement membrane in capillaries which subsequently results in haemorrhage [32]. They are known to induce systemic haemorrhage in addition to their effects on local tissues around the bite site. Hence, their effects on the ocular system, which is rich in blood capillaries (retina and choroid), are largely attributed to ocular haemorrhage and subsequent secondary inflammatory responses [14]. In addition, phospholipase A_2_ (PLA_2_) has been shown to induce anti-coagulant effects resulting in haemorrhage. Venom metalloproteases, PLA_2_ and L-amino acid oxidases can induce inflammatory responses [31]. Therefore, these activities may collectively induce ocular complications including optic neuritis. Cytotoxic three-finger toxins, often abundant in elapid venoms, can damage cell membranes [33] and may affect the integrity of the optic nerve. Similarly, PLA_2_ present in both elapid and viper venoms [34] are likely to directly affect the nerve membranes in the ocular system in addition to their wide range of neurotoxic effects. The membrane damage may lead to inflammatory responses which in turn affect the optic nerve. However, the optic nerve contains both myelinated sections and unmyelinated areas within the eye that fuse with the retinal cells. Hence, further research is required to underpin exactly how different venom toxins might directly interact with these types of neurons and lead to inflammatory responses resulting in optic neuritis. Neurotoxins present in elapid venoms can induce ocular muscle paralysis. The movement of four of the six eye muscles (ocular muscles) is controlled by the oculomotor nerve (also known as Cranial Nerve III). We note that both patients displayed attenuated pupil reaction to light which could be explained through the involvement of the sensory nerve (optic), or the motor response mediated by the motor nerve (oculomotor). There is much debate about the structure of the human ocular nerve with some suggesting that it is unmyelinated, whereas others show that the degree of myelination is low and uneven during early years but thereafter increases significantly with age [35]. Cadaver tissue profiling of the oculomotor nerve from people of the approximate age of our two patients has a clear sign of myelination. Hence, the abnormal reaction to light could in part be attributed to the demyelination of the oculomotor nerve as well as the optic nerve. Generally, SBE-induced posterior segment complications are attributed to direct actions of venom toxins mostly on blood capillaries, while anterior segment complications may arise because of secondary inflammation. While vascular complications are likely to occur following envenomation by vipers, due to the low abundance of proteolytic enzymes in elapid venoms, they are unlikely to induce such complications with high severity. Therefore, the underlying mechanisms in the development of optic neuritis following envenomations by krait and cobras should be further scrutinized to underpin the molecular relationship between their venom toxins and components of the ocular system. 

## 5. Conclusions

It is apparent that envenoming by any venomous snake species including elapids, vipers, and colubrids can induce optic neuritis. Hence, this condition, as well as other ocular manifestations, are not only attributed to typical envenomations of spitting cobras. The clinicians who treat SBE should be aware of this complication arising from non-spitting snake species specifically several days after the bite despite antivenom administration. Fundoscopic examination as well as CT and/or MRI scans together with thorough ophthalmological and neurological examinations can be used to identify optic neuritis. As reported in most cases, intravenous administration of corticosteroids may be effective in treating this inflammatory condition. Although optic neuritis upon envenomation by non-spitting cobras was reported previously, the development of this condition in a patient with krait envenomation emphasises the need for further research in this area. Moreover, the molecular interactions of various venom toxins with the optic nerves and other components of the eyes should be studied in detail to establish the underlying molecular mechanisms. Finally, the impact of antivenom components in the development of this condition should be investigated as this may be another potential cause which could be resolved through further refinements to antivenoms.

## 6. Materials and Methods

Both patients provided written informed consent to collect and publish the data presented in this article. The patients and their relatives were informed about the purpose of this study and its wider use in scientific and clinical communities to improve the clinical management of SBE. The identity of the offending snake species was confirmed by an experienced herpetologist by examining the dead specimens brought by the patients. The patients were diagnosed and treated as normal using standard protocols from the World Health Organisation and local health authorities. They received polyvalent antivenom raised against the Indian ‘Big Four’ snakes from Bharat Serum and Vaccines Limited, India. The diagnosis of optic neuritis was performed using standard ophthalmic and neurological examinations as well as using CT and MRI scanning equipment available within the hospital.

## Data Availability

Not applicable, as all data from this study are included within this article.
